# TAF1L promotes development of oral squamous cell carcinoma via decreasing autophagy-dependent apoptosis

**DOI:** 10.7150/ijbs.41148

**Published:** 2020-02-10

**Authors:** Daiwei Wang, Hong Qi, Haoxing Zhang, Wei Zhou, Yanpeng Li, Ang Li, Qiong Liu, Yun Wang

**Affiliations:** 1 Center for Research and Technology of Precision Medicine, College of Life Sciences and Oceanography, Shenzhen University, Shenzhen, Guangdong, China.; 2 Key Laboratory of Optoelectronic Devices and Systems of Ministry of Education and Guangdong Province, College of Optoelectronic Engineering, Shenzhen University, Shenzhen, Guangdong, China.; 3 Key Laboratory of Shaanxi Province for Craniofacial Precision Medicine Research, College of Stomatology, Xi'an Jiaotong University. Xi'an, Shanxi, China

**Keywords:** TAF1L, OSCC, apoptosis, autophagy, siRNA

## Abstract

This study focused on investigating the relationships of TAF1L expression and clinical features or pathological stages of oral squamous cell carcinoma (OSCC), and its potential roles of TAF1L on OSCC development. Western blot and immunohistochemical staining were used to detect TAF1L expression in OSCC tissues and cells. Effects of TAF1L on OSCC cells* in vitro* were examined by cell proliferation assay, wound healing assay, transwell chamber assay, flow cytometry analysis and siRNA technique. Cellular key proteins related to cell autophagy and apoptosis were evaluated by Western blot and immunofluorescent staining. Moreover, functions of TAF1L on OSCC process were observed in nude mouse model. Testing results showed that expression of TAF1L protein was higher in OSCC tissues than that in normal oral epithelial or paracancerous tissues. Additionally, the level of TAF1L protein expression was upregulated in OSCC cell lines, compared to that in normal oral epithelial cells. Furthermore, cell proliferation, migration, autophagy and apoptosis were modulated post siRNA-*TAF1L* treatment *in vitro*. Especially, TAF1L knockdown-induced apoptotic activation on OSCC cells could be rescued by autophagic activator (Rapamycin). Moreover, that overexpression of TAF1L protein could promote the growth of OSCC cell xenografts was confirmed in nude mouse model. Taken together, it suggests that TAF1L may facilitate OSCC cells to escape cell apoptosis via autophagic activation for enhancing OSCC development.

## Introduction

Oral squamous cell carcinoma (OSCC) is the most common carcinoma in human head and neck, which occurs approximately 90% of malignant tumors in oral cavity 1-3. Approximately 350,000 patients with OSCC are newly diagnosed each year, and two-thirds of those cases are detected in South East Asian 4,5. Although growing strategies of new treatments and diagnosis were generated over past decades, 5-year survival rate of OSCC has been less than 50% due to the many missed diagnosis and misdiagnosis at the early and middle stages of the tumor development, and detailed mechanism of OSCC remains unclear. Thus, to discover novel key biomarkers for early diagnosis, precision medicine, prognosis and intervention has been become a hotspot in OSCC researches.

TATA-box binding protein associated factors (TAFs) are required for transcription initiation with RNA polymerase II. TAF1 is a major member of the TAF family, and its function is as a scaffold for binding TATA-box binding protein and TAFs to TFIID 6-8. TAF1 like (TAF1L) is a TAF1 homologue, and till now, it has been found that both of them have a similar function with histone acetyltransferase activity 9,10. In cancer researches, although it has known that TAF1 can play important roles on cell proliferation and apoptosis 11, little is known in regards to that is pathophysiological functions of TAF1L. Our primary studies via bioinformatics, RNA-seq and multiple immunohistochemistry revealed that *TAF1L* gene with somatic mutations and overexpression, as an oncogene, could promote OSCC and esophageal cancer procession 12,13. Subsequently, growing studies have reported that deletions, point mutations, abnormal expression and inactivation of TAF1L were involved in the tumorigenesis of several cancers, such as lung, oral, gastric, colorectal, and urothelial cancers 14-17. However, more researches for the roles of TAF1L gene in tumorigenesis are still needed.

Cell apoptosis, one major cell death form, plays critical functions in the body development and disease process, especially involved in many cancers development process 18,19. Abnormal phenotype of TAF1 associated with cell apoptosis in cancers has been pointed out 20. In addition, the autophagy, another cell death form, also plays important roles in maintaining cellular homeostasis, nutrient stress, hypoxia stress, oxidative stress and mitochondrial damage 21,22. Occasionally, autophagic activation has been found to have the opposite effects in cancer development, according to tissue type and genotype 21,23-25. As known as the relationship between the autophagy and apoptosis is involved in some proteins, such as ATG3, ATG5, ATG7, Bcl-2, Beclin-1 and etc. 26-28. Recent researches indicated that the knockdown of those key genes associated with cell autophagy (such as ATG5, ATG7 and Beclin-1) could prevent the apoptosis 29,30. Several scientists have found that both cell autophagy and apoptosis were associated with the prognosis of OSCC 31-34.

In this study, based on the hypothesis that TAF1L abnormal expression may mediate a crosstalk of the apoptosis and autophagy during OSCC procession, we focused on investigating effects of TAF1L on tissues and cells of OSCC *in vitro* and* in vivo*, as well as on the autophagy and apoptosis of OSCC cell lines post siRNA-*TAF1L* and Rapamycin administration.

## Material and Methods

### Tissue collection

Two commercial tissue microarrays were purchased from Biomax (USA): one array (ID: OR208) included 60 sections of OSCC tissue and 9 sections of normal oral tissue (per tissue section for each case, total 69 cases), and another array (ID: OR601b) included 50 sections of OSCC tissue and 10 sections of normal oral tissue (same as one section per case, total 60 cases). In addition, 11 archived formalin fixed-paraffin embedded samples obtained from oral normal epithelial or paracancer tissues after acute injury repair or benign tumor resection were collected and served as normal controls. Total testing numbers were 110 cases of OSCC tissue and 30 cases of normal oral/paracancerous tissue were utilized as research objects in this study. Clinical parameters (e.g., gender, TNM classification, clinical stage, pathological grade, and etc.) of all cases individually accompanying with the two tissue microarrays were provided by the Biomax and listed in Table 1. Executive collection and treatment of the tissue samples in this study were approved by the Medical Ethics Committee of Shenzhen University.

### Immunohistochemical staining (IHC)

The tissue sections for IHC staining were firstly deparaffinized with xylene, and rehydrated with serial diluted alcohols. Sections with antigens were then retrieved in the egg-steamer with 10 mM citrate buffer (pH 6.0) at 100°C for 30 min, and followed the incubation with 3% H_2_O_2_ for 30 min, washed with PBS, and incubated with appropriate concentrations of primary antibody against special antigen at 4°C, overnight. After washing with PBS again, sections were incubated with secondary antibody (goat anti-rabbit IgG-HRP, Zymed Laboratories, USA) for 60 min at room temperature. Positive signals were showed up by using AEC kit (GBI, USA), and nuclear counterstaining was done with hematoxylin. PBS replaced with primary antibody was served as negative staining. Anti-Ki67 antibody (Rabbit, 1:2000, Proteintech, USA), replacing the primary antibody, was selected as the positive control for checking the staining procedure. To set an immunopositive control, appointed tissue sections, which colorectal cancer tissue sections were selected in this study, were detected as corresponding to primary antibody against specific antigen. Immunoreactive scores (IRS) were assessed with positive percentage of total cells and positive intensity of mean obtained from 5 areas per section randomly under a microscope at 400 × magnification 35,36. Staining scores were achieved by two examiners independently, and showed a score consistently was > 82%. If the image record was with different opinion from two examiners, the re-evaluation would be made by a third one. The degrees of positive signal intensity were determined using the following scale: 0 - none, 1 - weak, 2 - moderate, 3 - intense and 4 - strongly intense. Percentages of positive cell were counted as (0) 0%, (1) 1-25%, (2) 26-50%, (3) 51-75% and (4) 76-100%. Final staining score was achieved by multiplying two scores of the degree of positive signal intensity and the percentage of positive cell. A score of < 2 was considered as “negative” and ≥ 2 as “positive”, and the categories were used for statistical analysis.

### Antibodies and reagents

Antibodies used in this study were obtained from (1) Proteintech (USA), including rabbit pAb against TAF1L, rabbit pAb against LC3B, rabbit pAb against Bax and rabbit pAb against P62/SQSTM1; (2) Cell Signaling Technology (USA), including rabbit pAb against GAPDH, rabbit pAb against Caspase-3 and mouse mAb against Bcl-2; (3) Abmart (USA), mouse mAb against GFP; and (4) BD Biosciences (USA), secondary antibodies. The Alexa-conjugated secondary antibodies were from BD Biosciences (USA). In addition, the Rapamycin, an autophagic activator, was purchased from Solarbio (USA).

### Cell culture and transfection

Four cell lines from oral tissues (HOEC, DOK, Tca-8113 and Ca9-22) were seeded at an initial density of 5×10^4^/cm^2^ in collagen precoated 6-well plates with RPMI-1640 medium or DMEM/Ham's F12 medium supplemented containing 10% fetal bovine serum, and incubated at 37°C with 5% CO_2_. When growing up to approximately 70% confluence, cells were transfected with LC3-GFP plasmid (a kind gift from Dr. Jieli Wei, University of Chinese Academy of Sciences, China) and Lipofectamine 2000 (Invitrogen, USA). For siRNA transfection, cells were transfected using siRNA*-TAF1L(s)* and siRNA-negative control at 100 nM concentration. Three reconstructed vectors of *TAF1L* gene silencing were generated with 3 pairs of sequencing primers (including sense and anti-sense primers), which were synthesized by Sangon Biotech (China), and listed as followed: TAF1L-siRNA#1: 5'-GACCCAACAACCCUUCAUTT-3' and 5'-AUGAAGGGUUGUUUGGGUCTT-3'; TAF1L-siRNA#2: 5'-GGAAGACUCUGAUGUGGAUTT-3' and 5'-AUCCACAUCAGAGUCUUCCTT-3'; TAF1L-siRNA#3: 5'-GGAUGGGAAACCUAAGCCUTT-3' and 5'-AGGCUUAGGUUUCCCAUCCTT-3'; NC-siRNA: 5'-UCUCCGAACGUGUCACGUTT-3' and 5'-ACGUGACACGUUCGGAGAATT-3'. After 48 hr transfection, cells were treated for evaluating cell functions. To measure the efficacy of siRNA*-TAF1L* in transfected cells, expression levels of candidate protein were also analyzed by Western blot.

### Rapamycin treatment

Each Ca9-22 or Tca8113 cell line was divided into two groups based on siRNA-*TAF1L* or siRNA-control treatment, and then each cell group was administrated with 0.1 μM Rapamycin (Rapa) or same diluent (as negative control) for 16 hr. The cellular effects on candidate proteins of apoptosis and autophagy after Rapamycin administration were evaluated via Western blot.

### Generating stable TAF1L protein overexpression cells

To establish stable TAF1L protein overexpression in OSCC cells, full length coding region of human *TAF1L* gene was subcloned into the pLV3-IRES-puro vector. And then, the TAF1L-pLV3-IRES-puro vectors were packaged into viral particles in HEK293T cells. When re-constructed Tca-8113 cells were selected as a stable TAF1L protein overexpression cell model, those cells were again treated with 0.5 μg/ml Neomycin for two weeks.

### CCK-8 cell proliferation assay

Tca-8113 and Ca9-22 cells were seeded into 96-well culture plates with 3 × 10^3^ cells per well. At each scheduled time point, a mixture of 100 μl fresh medium and 10 μl CCK-8 (MCE, USA) was added per well, and plates with cells were incubated at 37°C for 1 hr. The absorbance of CCK-8 was detected at 450 nm using a microplate reader (BioTek, USA).

### Wound healing assays

Tca-8113 cells and Ca9-22 cells were seeded in 12-well culture plates at a density of 1×10^5^ per well. When cells grew up to ~ 90% confluence, cell wound was created by scratching with a 200 μl-pipet tip. The cells were then washed with PBS, incubated with fresh media, and taken the photograph under a microscope, in order to observe scratching wound at 0 hr-start time point and at 48 hr-wound healing time point. The cell migration was assayed by measuring the distance between two edges of scratched wound at above pointed 0 and 48 hr with Image-Pro Plus 6.0.

### Transwell chamber assay

Cellular invasion capacity was examined using a 24 well Costar Transwell Chamber System with Matrigel matrix (BD Biosciences), according to the manufacturer's protocol. 2 × 10^4^ cells per well were seeded into the upper chamber of this system with 0.5 ml of fresh medium containing 1% FBS, and the lower chamber was filled with same volume of medium containing 15% FBS, and then cells were incubated at 37°C in 5% CO_2_ for 24 hr. After incubation, the filter membrane between the upper and lower chambers were fixed with the methanol, the cells remaining on the upper surface of the membrane were removed with a cotton swab, and the cells at lower surface of the membrane were stained with 0.5% crystal violet for 15 min. The cells were finally recorded in 5 fields randomly under a microscope (200 × magnification), and numbers of invading cell were counted by Image-Pro Plus 6.0.

### Apoptosis detection with flow cytometry

Tca-8113 and Ca9-22 cells were stained with FITC Annexin V Apoptosis Detection Kit II (BD), according to the manufacturer's protocol. After 48 hr siRNA*-TAF1L* transfection, 1 × 10^6^ cells/ml of Tca8113 cells or Ca9-22 cells were washed with cold PBS, resuspended with binding buffer, incubated with 5 μl Annexin-V-FITC reagent (positive signals can indicate early apoptosis) at room temperature for 5 min, and stained with 10 μl PI reagent (positive signals can indicate late apoptosis) at room temperature for 5 min. The percentages of apoptotic cell were analyzed by FACS Calibur flow cytofluorometry (BD).

### Western blot

Total cellular proteins were collected after different treatments and cultured in 6-well planes with RIPA lysis buffer (Biosharp, USA) containing protease cocktail (Roche, Germany) for 30 min at 4°C. After the centrifugation at 14,000 × g for 10 min at 4°C, each cellular lysate was transferred to a new 1.5 ml centrifuge tube on ice, and protein concentration was determined with the BCA assay (Thermo Scientific, USA). For Western blot analysis, equal amounts (20-30 μg) of cell protein were loaded on SDS polyacrylamide gels for electrophoresis, and transferred to nitrocellulose membranes (Whatman, Germany). The membranes were blocked with TBST contained 5% BSA for 30 min at room temperature. Candidate proteins were identified with specific primary antibodies at 4°C, overnight, and followed the incubation with horseradish peroxidase (HRP)-conjugated secondary antibody for 1 hr at room temperature. The bands were visualized and measured via the ECL kit (Pierce, USA) and Tanon analysis system (Tanon, China).

### Immunofluorescent staining

Tca-8113 cells and Ca9-22 cells were grown on precoated coverslips, and fixed in 2% PFA in phosphate-buffered saline (PBS) for 15 min at 37°C. Cells were blocked with 3% BSA in PBS for 30 min at room temperature for non-special staining, and cells were then incubated with primary anti-GFP antibody (1:500) and secondary antibody (Alexa Fluor 488, 1:500) for 1 hr at room temperature orderly. For recording positive fluorescent signals of GFP puncta (green Color), an Olympus FV1000 microscope was used. The fluorescent images were taken with a 60 × oil-immersion lens.

### Nude mice of OSCC

Female nude mice (6 week-old, BALB/-nu) were divided into two groups randomly (n ≧ 5 for each group), and treated with the transfection of TAF1L-stable expression cells (Tca8113-TAF1L cells) or untreated control cells (Tca8113-control cells). The prepared cells were suspended in IMDM medium without serum, and a concentration at 6×10^6^ cells was subcutaneously injected in one mouse back to establish xenogenetic model *in vivo*. The subcutaneous tumor growth was measured with a Vernier caliper every other day. The formula (length × width^2^/2) was served as for calculating tumoral volume. The animal tests for establishing nude mouse model were approved by the Experimental Animal Ethics Committee of Shenzhen-PKU- HKUST Medical Center.

### Statistical analysis

All experimental data obtained from this study were performed with at least three biological repeats, except of the nude model. Error bars in the figures were represented Mean ± SD. Statistical analyses were performed by using SPSS 19.0, Graphpad prism 6 and Excel (Microsoft) with Student's *t*-test, one-way ANOVA, and Fisher's exact test. *p* < 0.01 was considered as significant difference.

## Results

### Overexpression of TAF1L was associated with OSCC process

**Figure 1:**
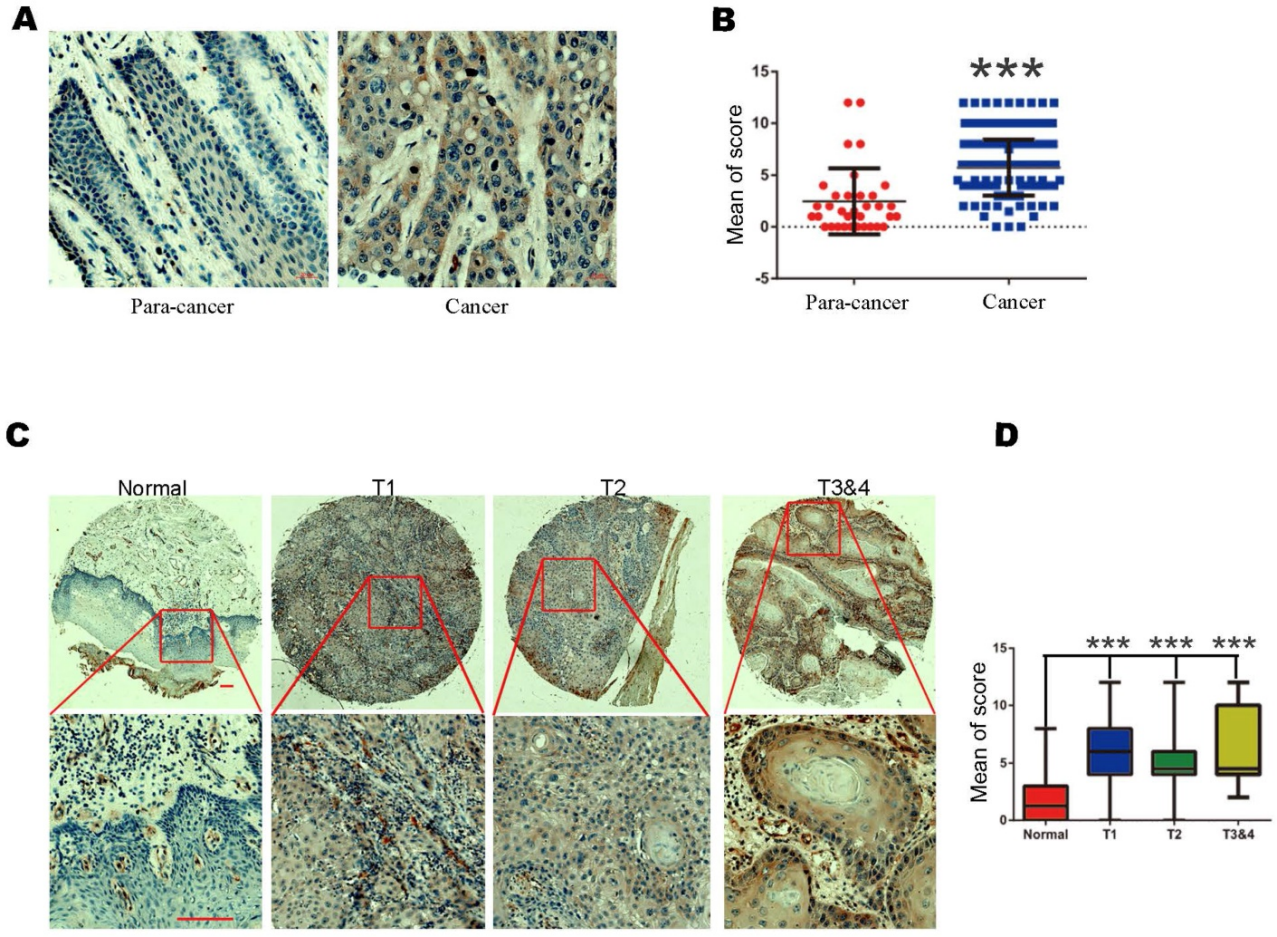
The levels of TAF1L protein expression in OSCC tissues and normal oral/paracancerous tissues were observed by IHC. A, TAF1L overexpression in OSCC tissues vs. TAF1L lower expression in normal tissues. Scale bars: 20 μm. B, Quantification of the mean scores of TAF1L positive signals between OSCC tissues and normal oral epithelial tissues. C, Overexpression of TAF1L in OSCC tissues was showed at different TNM-T stages, compared with normal oral epithelial tissues. Brown color showed positive signals. Scale bars: 20 μm in the top row and 100 μm in the bottom row. D, The means of density scores of TAF1L were showed higher at different TNM-T stages of OSCC, compared with normal oral epithelial tissues. OSCC tissues: n = 110, normal oral/paracancerous tissues: n = 30. Positive signals were showed with brown color. *** *p* < 0.0001 (one-way ANOVA).

To observe the changes of TAF1L protein expression in OSCC procession *in situ*, IHC staining was performed on a total of 140 formalin fixed-paraffin embedded tissue cases, which included 110 cases of OSCC tissues and 30 cases of normal oral epithelial/paracancerous tissues. Positive TAF1L signals by IHC staining were showed that mainly distributed in the membrane and cytoplasm of cancerous epithelial cells and a few of infiltrating cells (Figure 1A). Comparatively, TAF1L signal intensity in OSCC tissue sections was much stronger than those in normal oral or paracancerous tissue sections (Figure 1A, B). Up to 87.3% (96 positive of total 110) expressed strong positive signals in OSCC tissues, on the contrary, only 28.1% had positive signals in normal oral or paracancerous tissues (9 positive of total 30). The difference between above two groups was statistically significant (Figure 1B; Tables 1 and 2; *p* < 0.001).

Then, the relationships between TAF1L protein expression in OSCC tissues and clinical stage, differentiation grade or TNM stage of this disease were next investigated. Imaging results showed that the levels of TAF1L protein expression were significantly increased at all clinical and pathological phases of OSCC (Figure 1C, D; Table 3). Using the chi-square and Fisher's exact analyses, similar results were shown in the comparison of TAF1L protein expression at any two stages from differentiation grade I-II to III-IV and TNM T1-2 to T3-4. In addition, the association of the level of TAF1L protein expression with clinical or pathological feature of OSCC procession was also analyzed, and the results were listed in Table 3.

### Cell proliferation of OSCC was suppressed by *TAF1L* gene depletion

**Figure 2:**
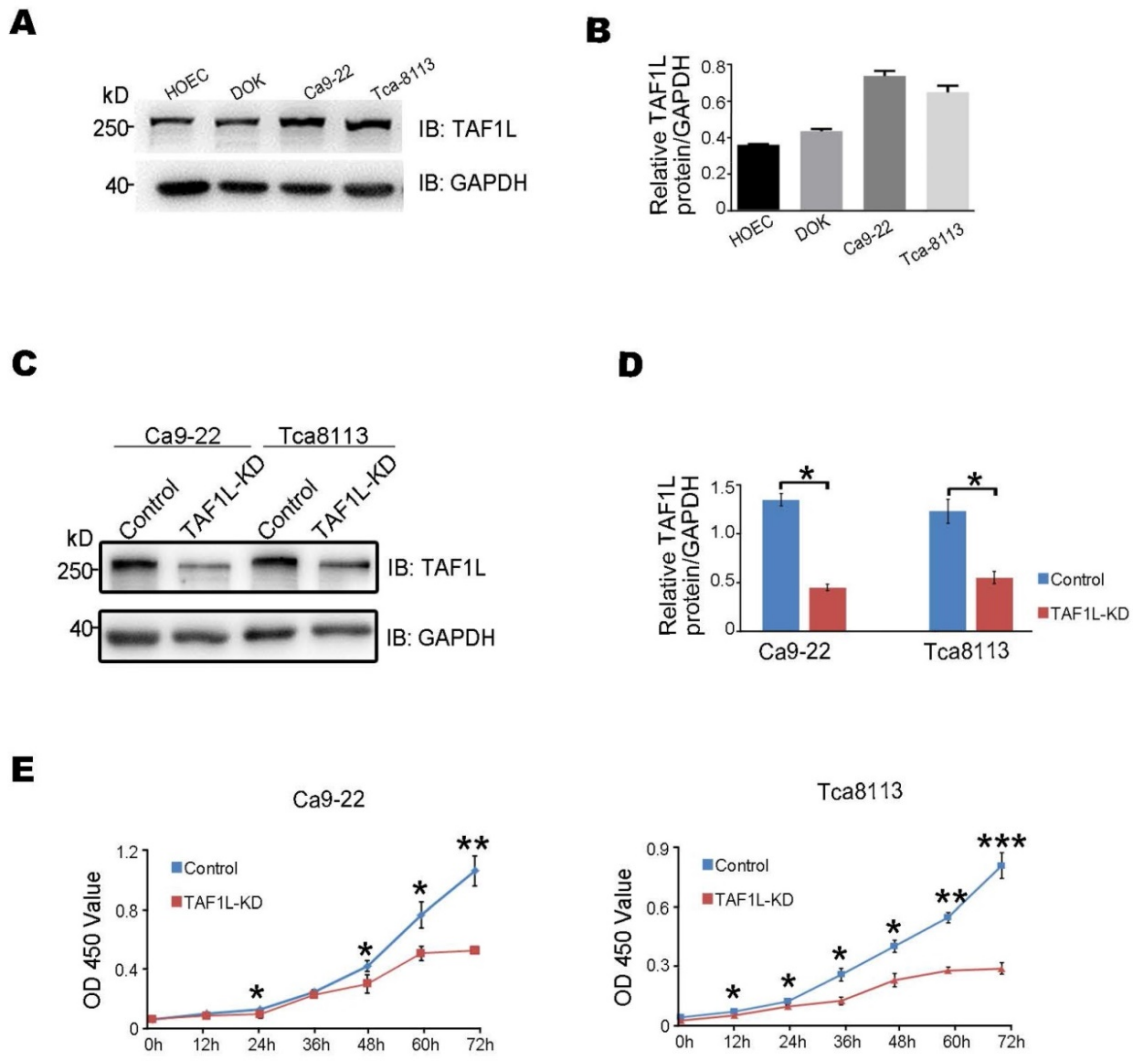
The levels of TAF1L protein expression of OSCC cells after *TAF1L* gene knockdown with siRNA technique were detected by Western blot and CCK-8 assays. A, TAF1L protein expression of a panel of OSCC cells was showed with special antibody against TAF1L antigen. B, TAF1L protein level related to (A) was measured based on the intensity of Western blot. C, Cells were detected after 48 hr transfection with selected siRNA-*TAF1L* or siRNA-control. Verification of *TAF1L* gene knockdown post siRNA treatment in Ca9-22 cells or Tca-8113 cells was via Western blot with antibody against TAF1L or GAPDH. D, Downregulation of TAF1L was tested at the protein level related to (C). E, Using the CCK-8 assay and cell proliferation were measured in Tca-8113 cells and Ca9-22 cells after siRNA-*TAF1L* (TAF1L-KD) or siRNA-control transfection for time response. Cell viability was determined at scheduled time point. Data were showed Mean ± SD, N = 3, ^*^p < 0.01, ^**^p < 0.001, ^***^p < 0.0001 (Student's *t*-test).

As the expression of TAF1L protein was such higher in OSCC lesion (Figure 1), it seems to indicate that TAF1L may play important roles in cellular pathology of OSCC. Thus, the detection of TAF1L expression was performed in a panel of OSCC cell lines, such as Ca9-22 (cancerous gingival epithelia) and Tca-8113 (tongue cancer) cells, as appropriate cell lines for further testing. And they were compared with that expression in HOEC (normal oral epithelial cells) and DOK (dysplastic oral keratinocytes) by Western blot. From this test, the levels of TAF1L protein in both Ca9-22 and Tca-8113 OSCC cell lines were shown to have higher expression than that in normal cell line and DOK cell line (Figure 2A, B). Therefore, these two OSCC cell lines were chosen for transfecting siRNA-*TAF1L* and siRNA-control to assay the effects of TAF1L on OSCC cells *in vitro*. Then, cell proliferation in OSCC cell lines was examined with CCK-8 cell proliferation assay. Both growth rates of Ca9-22 and Tca-8113 cells were decreased after siRNA*-TAF1L* treatment at 72 hr (both *p* <0.001; Student's *t*-test; Figure 2C-E). Those results showed that TAF1L gene silence could impede the growth of OSCC cells.

### The migration and invasion of OSCC cells were reduced after* TAF1L* gene depletion

**Figure 3:**
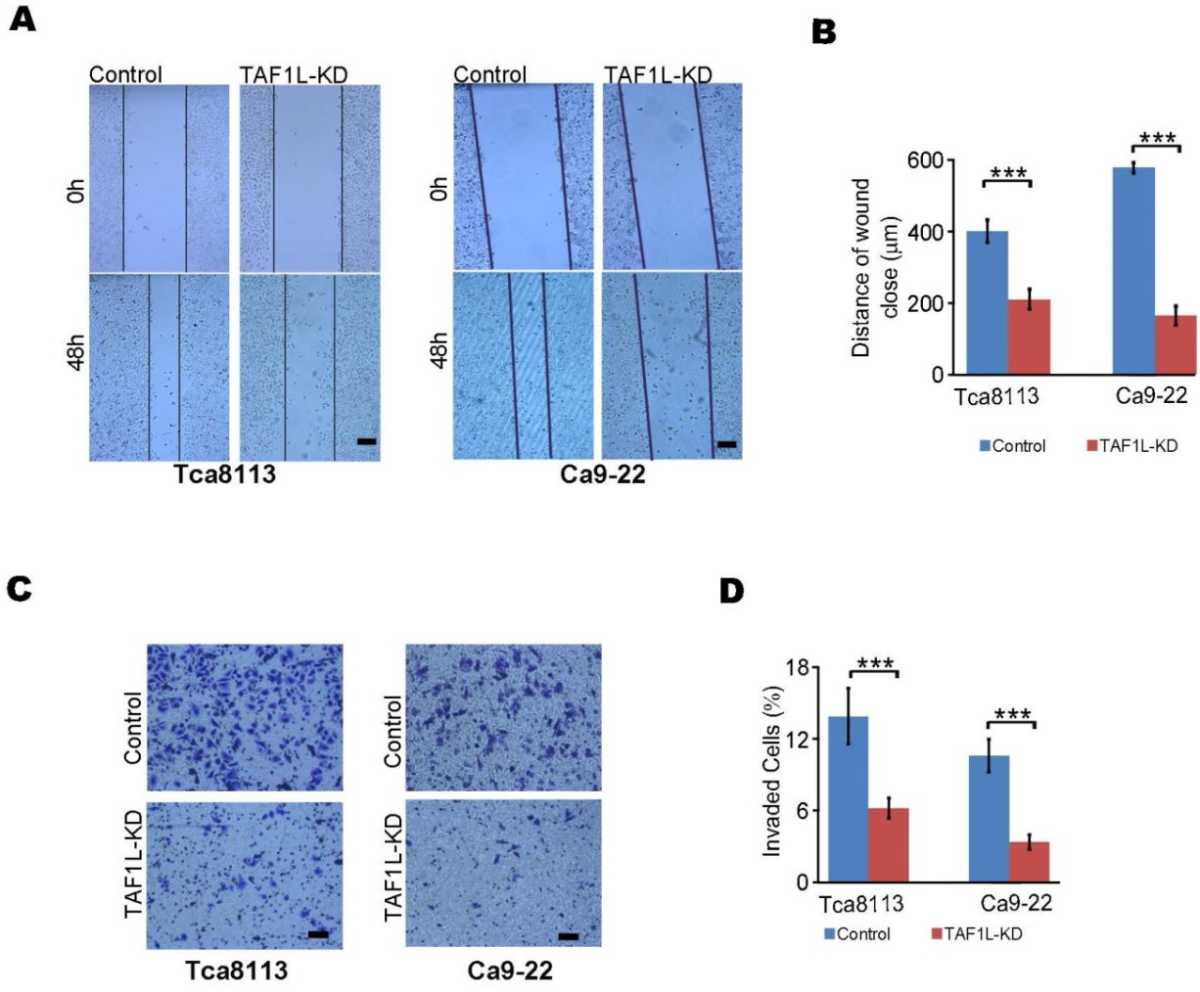
The migration and invasion of OSCC cells were decreased by siRNA-*TAF1L* treatment. A, Cell migration into the open space was monitored. Scale bars: 100 μm. B, Quantification of the length of migration was generated at appoint time after wounding was made from (A). C, Cell numbers for measuring the invasion were counted via transwell invasion assay. Scale bars: 100 μm. D, Those invading cells were counted, and compared with those obtained from (C) for quantified analysis. Scale bars: 100 μm, Student's *t*-test: Mean ± SD, N=3, **p < 0.001, ***p < 0.0001.

To investigate effects of TAF1L on cell migration and invasion of OSCC by siRNA technique, the results of wound healing and transwell assays were analyzed in TAF1L gene knockdown cells of Tca-8113 and Ca9-22. Migration distance was markedly decreased in siRNA*-TAF1L* group (218.44 ± 14.86 μm), compared with that in siRNA-control group (415.31 ± 31.87 μm) (*p* = 0.00088). The same trend of that change was also observed in Ca9-22 cells, in which migrated distance was 170.31 ± 27.22 μm in siRNA*-TAF1L* group and 498.50 ± 29.01 μm in siRNA-control group,* p* = 6.58511E-06 (Figure 3A, B). Similarly, the numbers of invading cells were significantly suppressed in siRNA*-TAF1L* groups (5.78 ± 0.79%, 3.18 ± 0.55%), compared with that in siRNA-control groups (13.04 ± 2.22%, 9.91 ± 1.29%) in both OSCC cell lines (*p* < 0. 01; Figure 3C, D). Those data showed that migratory and invasive abilities of OSCC cells could be inhibited via TAF1L gene silence.

### The upregulated apoptosis of OSCC cells was induced by *TAF1L* gene depletion

**Figure 4:**
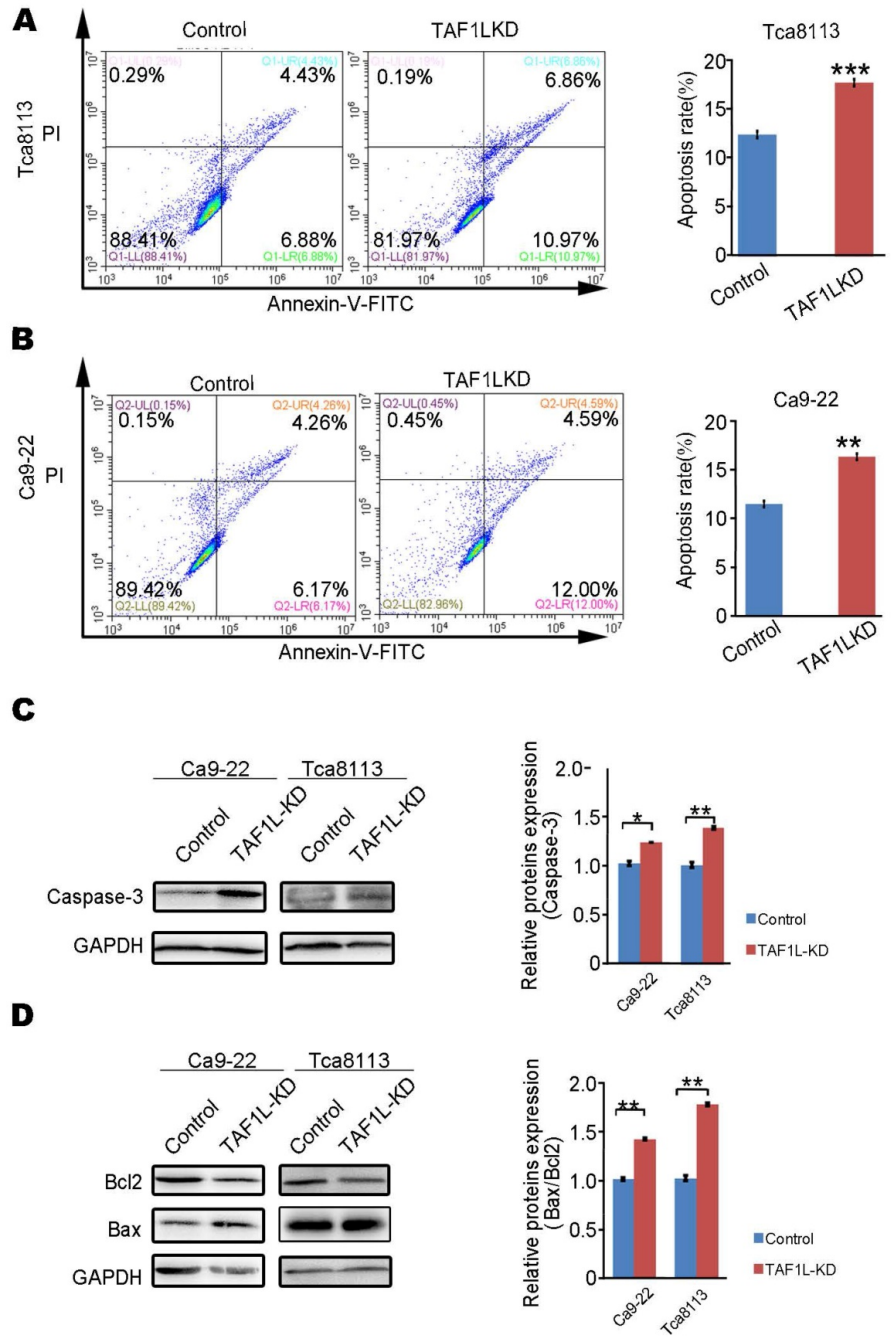
Apoptosis of OSCC cells were evaluated by *TAF1L* gene silence. A and B: The rates of cell apoptosis were observed in Tca-8113 cells (A) and Ca9-22 cells (B) transfected siRNA-*TAF1L* (*TAF1L*-KD) under a flow cytometer. C: Effects of *TAF1L* gene knockdown on cell apoptosis related Caspase-3 protein in Tca-8113 cells and Ca9-22 cells were detected with Western blot. D: Effects of *TAF1L* gene knockdown on apoptosis related Bax and Bcl-2 proteins in Tca-8113 and Ca9-22 cells were evaluated with Western blot. Protein quantification: Mean ± SD, N = 3, ^*^p < 0.01, ^**^p < 0.001 (Student's *t*-test).

In order to identify whether TAF1L expression can affect the apoptosis of OSCC cells, assays of flow cytometry and Western blot were performed to evaluate cell apoptosis in Tca-8113 cells and Ca9-22 cells post *TAF1L* gene depletion via siRNA-*TAF1L*. In results of flow cytometry, the ratios of apoptotic cells (including early apoptotic cells ratio + late apoptotic cells ratio) in both Tca-8113 and Ca9-22 cells were increased in siRNA*-TAF1L* treated groups (17.445 ± 0.272 % and 15.117 ± 1.190 %), compared with that in siRNA-control groups (12.555 ± 0.257 % and 11.710 ± 0.184 %), (*p* <0.001; Figure 4A, B). Then, classical protein markers of apoptotic proteins (e.g., Caspase-3, Bcl-2, and Bax) were also evaluated by Western blot. As expected, the levels of key proapoptotic proteins (Caspase-3 and Bax) were upregulated, and Bax/Bcl-2 ratio was also increased in siRNA-*TAF1L* treated groups, compared with that in siRNA-control groups in both Tca-8113 and Ca9-22 cells (*p* < 0. 01; Figure 4C, D). Those results indicated that apoptosis of OSCC cells could be significantly induced by *TAF1L* gene depletion.

### The autophagy of OSCC was inhibited by *TAF1L* gene depletion

**Figure 5:**
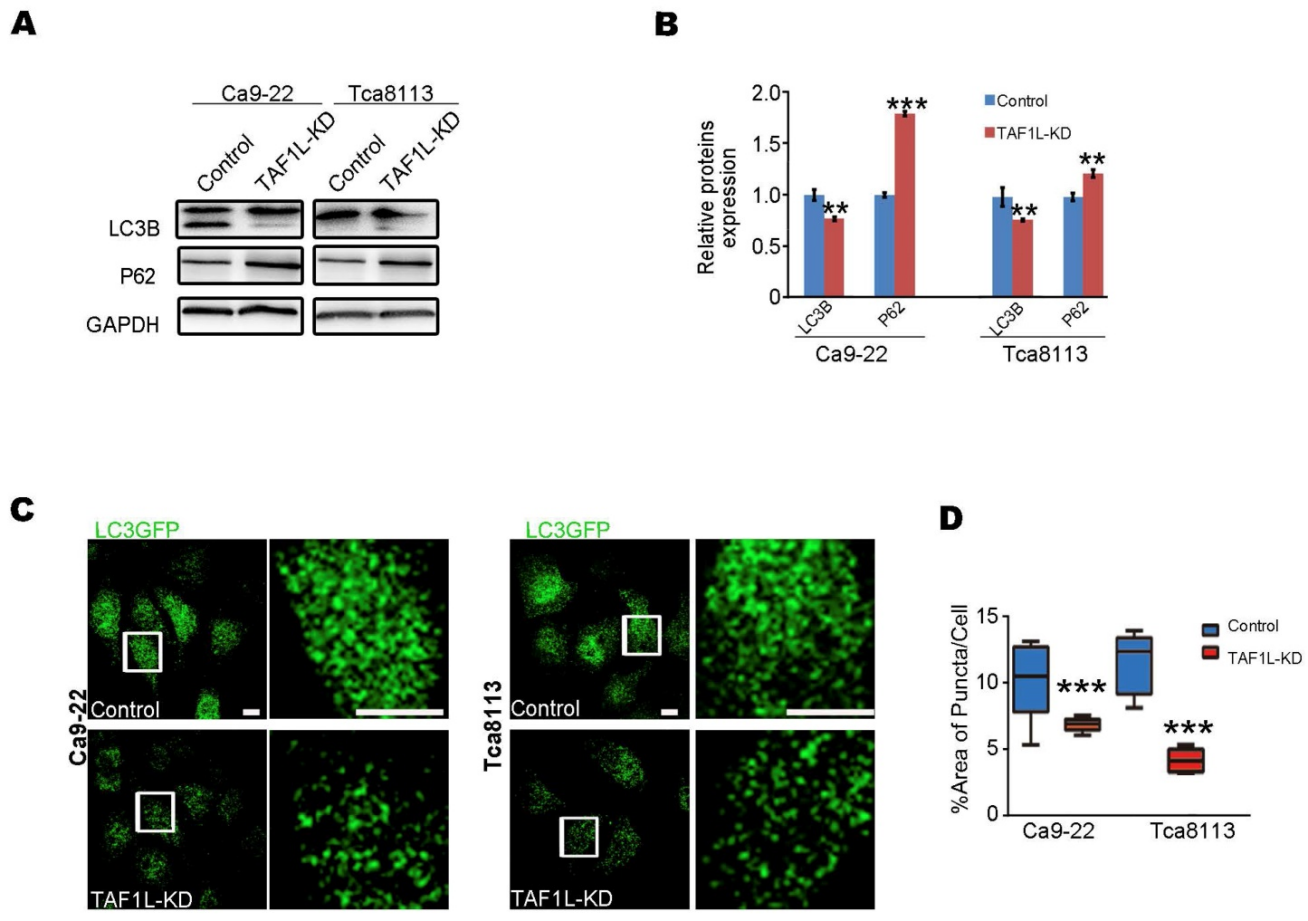
Effects of *TAF1L* gene depletion on cell autophagy were showed in OSCC cells. A, Autophagy-related proteins (LC3B and p62) were detected post *TAF1L* gene knockdown by Western blot. B, Proteins quantification was related to (A). C,** A**utophagic activity was assayed via confocal microscopy with immunolabeling for autophagy-related LC3B-GFP (green color). Scale bars: 5μm. D, Quantification of the ratio of green dot area (LC3B-GFP) to total cell area in (C). >30 cells per condition from three independent experiments were analyzed. Mean ± SD, N = 3, ^*^p < 0.01, ^**^p < 0.001, ^***^p < 0.0001 (Student's *t*-test).

In order to evaluate the effects of *TAF1L* gene on autophagy of OSCC cells by the gene knockdown, Western blot and Immunofluorescent staining assays were carried out in Tca-8113 and Ca9-22 cells. Firstly, the levels of key protein expression related autophagy (e.g., LC3B and p62) were detected by Western blot. Then the level of LC3B protein expression was downregulated and the level of p62 protein expression was upregulated in both Ca9-22 cells and Tca-8113 cells after siRNA*-TAF1L* treatment, compared with that in normal cells (*p* < 0.01; Figure 5A, B). To confirm their effects, LC3-GFP plasmid was further transfected for analyzing autophagic effects on OSCC cells. As shown in Figure 5C, the numbers of LC3-GFP punctum decreased when OSCC cells were treated with siRNA-*TAF1L*, compared with that in control cells (*p* < 0.01; Figure 5C, D). Above results demonstrated that depletion of *TAF1L* could inhibit cell autophagy of OSCC.

### The autophagy-depended apoptosis in *TAF1L* gene depleted-OSCC cells was verified by Rapamycin administration

**Figure 6:**
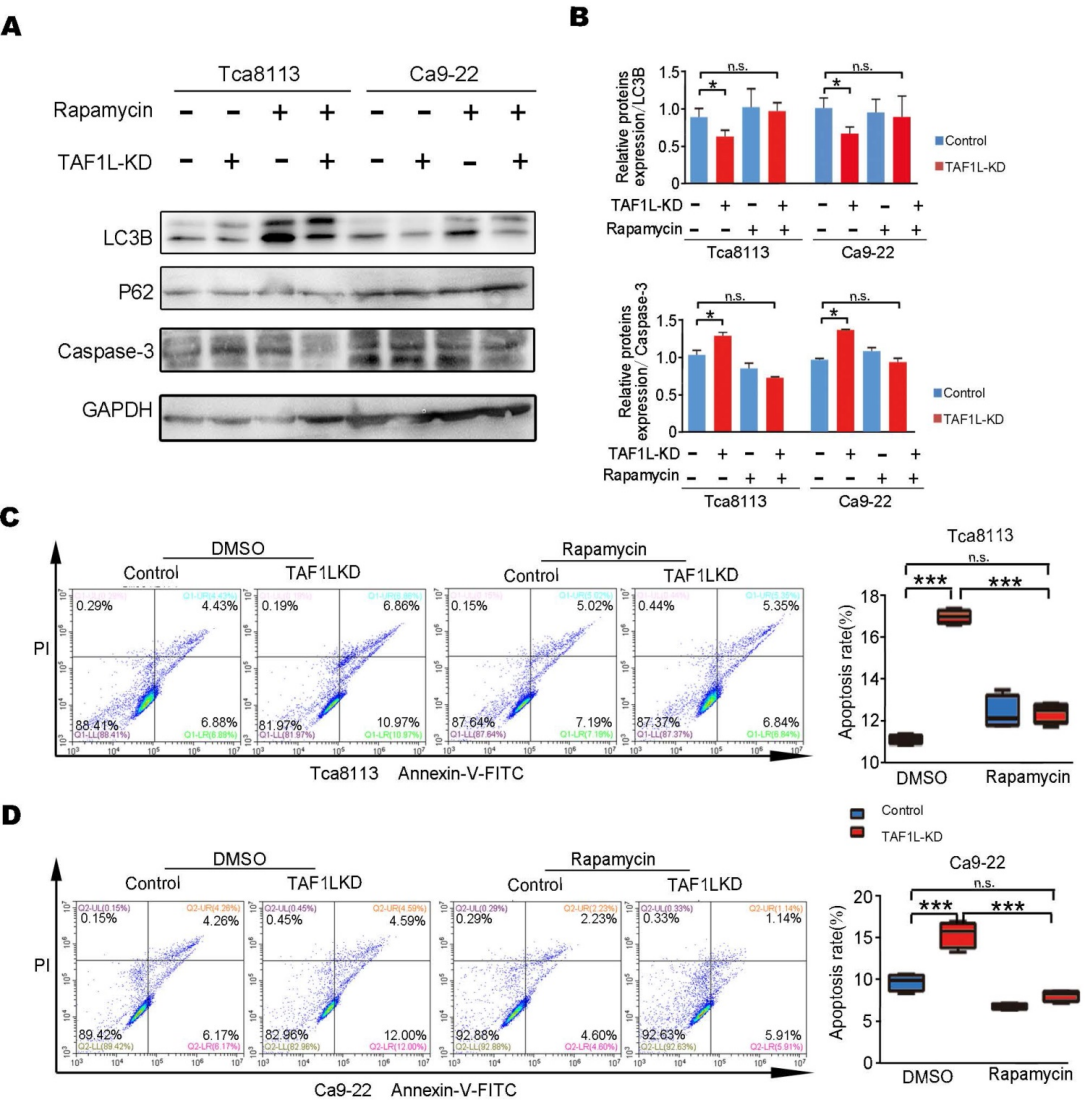
*TAF1L* gene silence-induced apoptosis in OSCC cells could be rescued by the Rapamycin treatment. The Ca9-22 and Tca8113 cells were divided into two groups based on siRNA-*TAF1L* or siRNA-control treatment, and then treated with 0.1 μM Rapamycin (Rapa) or the diluent with same concentration of dimethyl sulphoxide (DMSO) for 16 hr. A, Autophagy- and apoptosis-related markers (e.g., LC3B, p62 and Caspase-3) were tested with Western blot. B, Levels of protein expression in (A) were shown up (Mean ± SD, N = 3, ^*^p < 0. 01, one-way ANOVA). C-D, By flow cytometry, the image on the left showed changes of apoptotic cell ratios in Tca-8113 cells (C) and Ca9-22 cells (D), and on the right one showed percentages of apoptotic cell in the same cell line. Mean ± SD, N = 3, *p < 0.01, **p < 0.001, ***p < 0.0001 (Student's *t*-test).

The relationship between the autophagy and apoptosis was clarified in *TAF1L* gene depleted-OSCC cells. Since early data from this study showed that *TAF1L* gene depletion might inhibit autophagy in OSCC cells, Rapamycin 37, a well-known autophagic activator, was used to reveal the effects of *TAF1L* on both Tca-8113 cells and Ca9-22 cells via that gene knockdown. By contrast, results from Western blot showed that the level of LC3B protein expression was increased, while the level of p62 protein expression was decreased after Rapamycin administration. Those data points seem to indicate that the autophagic activation of Tca-8113 and Ca9-22 cells could be resulted in Rapamycin treatment. In addition, after treating 0.1μM Rapamycin for 16 - 24 hr, the expression of Caspase-3 was also reduced (Figure 6A, B). Intriguingly, ratios of apoptotic cell in both Tca-8113 and Ca9-22 cell lines were directly decreased by Rapamycin, when *TAF1L* gene expression was suppressed (Figure 6C, D). However, cells treated with Rapamycin alone was failed to affect cell apoptosis in this system. So that with the feedback for autophagic activation might be as a driver for apoptotic decrease in *TAF1L*-depleted OSCC cells. Results in this study indicated that *TAF1L* gene overexpression could reduce the apoptosis through the inhibition of the autophagy in both Tca-8113 and Ca9-22 cells.

### TAF1L protein expression enhanced tumor growth in OSCC *in vivo*

**Figure 7:**
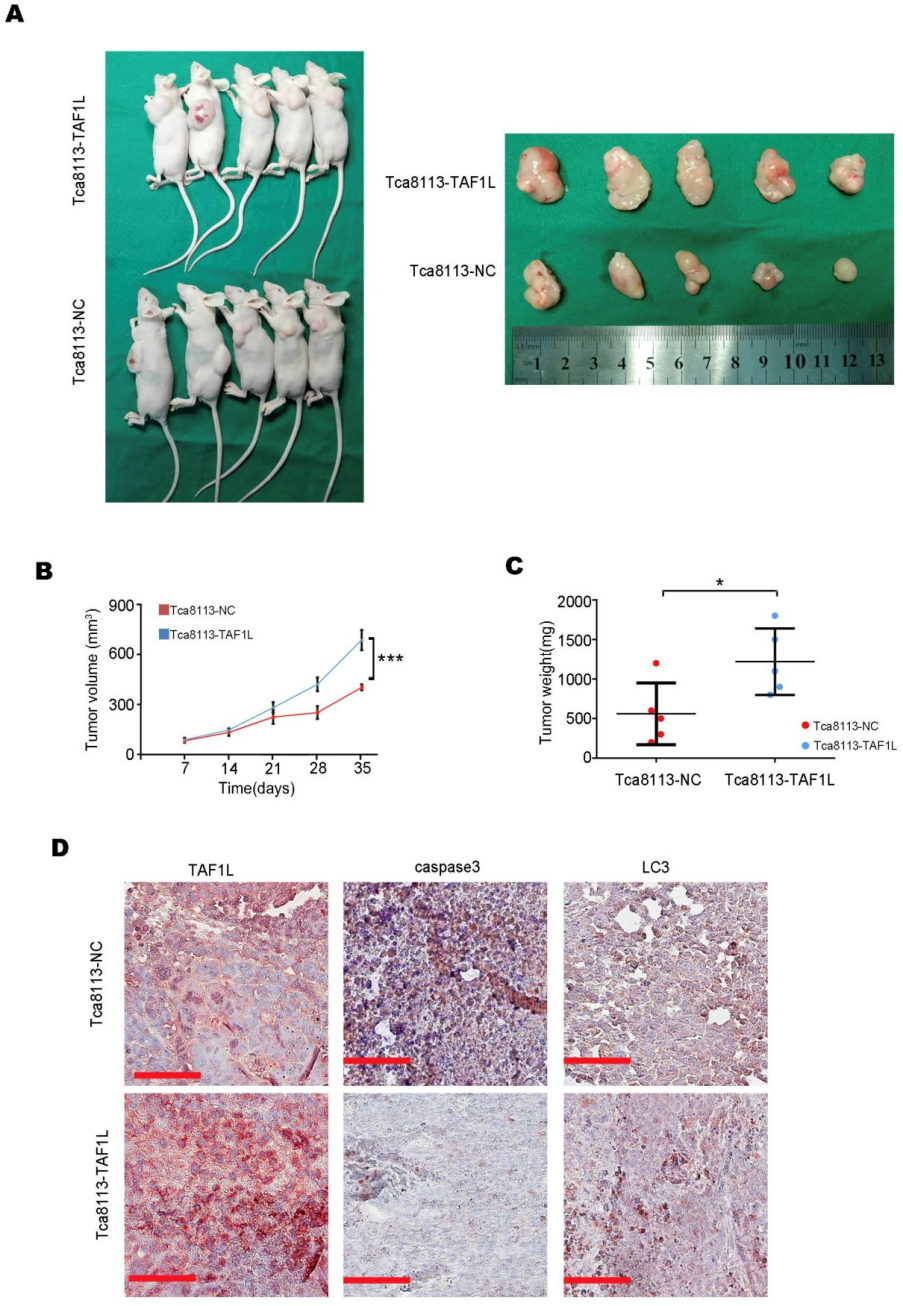
TAF1L overexpression promoted OSCC tumor growth* in vivo*. A, Female nude mice (left) and tumor xenograftes (right) were represented at 35 days post subcutaneous injection. B, The curves of tumor volume in nude mice were showed up (^***^p < 0. 001, Student's *t*-test). C, The curves of tumor weight were represented at 35 days post subcutaneous injection, ^*^p < 0.05 (Student's *t-*test). D, Comparison of TAF1L, apoptosis and autophagy markers expressions in the xenograft model. Scale bars: 100 μm.

To evaluate the effects of TAF1L protein overexpression on tumor growth of OSCC *in vivo*, Tca-8113-TAF1L cells with the clone expressed TAF1L protein stably or Tca-8113-NC cells with the clone expressed negative control protein stably (as negative controls) were injected into the back of nude mice with subcutaneous route. The results showed that the xenograft volumes were obviously increased in the Tca-8113-TAF1L group, compared with that in the Tca-8113-NC group (Figure 7A, B). The tumor weight was also slightly enhanced in Tca-8113-TAF1L group, compared with that in Tca-8113-NC group (Figure 7C). The correlation among *TAF1L* gene, apoptosis and autophagy related markers in tumor lesions from mouse model was verified by using IHC. Images of IHC staining demonstrated autophagic marker - LC3 was upregulated slightly, whereas apoptotic marker - Caspase 3 was downregulated in tumor tissues post TAF1L - overexpression treatment *in vivo* (Figure 7D). These findings mean that overexpression of TAF1L could promote tumor growth of OSCC *in vivo*.

## Discussion

A majority of OSCC cases are associated with high rate of incidence, recurrence, deterioration and death, and with the 5-year survival rate at lower level when they diagnosis at middle and late phases 38,39. In order to find novel biomarkers for early diagnosis and precision treatment, many mutations in some genes, such as *FAT1, TP53, CDKN2A, CASP8* and *NOTCH1,* were more frequently detected in OSCC patients by whole exome sequencing 40. However, the relationship between their effects on pathological procession and clinical outcome of OSCC patients need to be clearly studied. Meanwhile, for understanding the molecular mechanism of OSCC is also significant to benefit effective intervention of OSCC development.

Currently, there is little known regarding the biological functions of TAF1L, especially its pathological effects on cancer. Up until now, only a few research studies have reported that TAF1L mutation is associated with cancer pathogenesis. For example, a current study found that recurrent mutations of TAF1L gene were correlated with the pulmonary carcinoid tumor 14. TAF1L frameshift mutations were found in gastric and colorectal cancers due to mononucleotide repeats 16. Andrea et al. displayed that missense mutations in TAF1L gene were observed in advanced urothelial cancer 17. However, its mechanism and effects on cancer development remain obscure. In previous study, we used RNA-seq in mutation screening, and identified TAF1L as both significantly mutated gene and tumor-specific disruptive gene in OSCC 13. The same trend was found in the Japanese population, the numbers of somatic mutation of TAF1L in OSCC was approximately 10.6% 15. This study intended to reveal relationships of TAF1L expression with clinical features or pathological stages of oral squamous cell carcinoma. Via the image results of IHC staining, TAF1L signal intensity in OSCC tissue sections was much stronger than those in normal oral/paracancerous tissue sections. Comparatively, ≥ 87.3% strong positive signals of TAF1L protein was in OSCC tissues and only ≤ 28.1% weak positive signals of that was in normal oral and paracancerous tissues. Additionally, TAF1L protein was abnormally overexpressed in OSCC lesions at all clinical and pathological phases, even if it was at very early phase, and it was also showed more overexpression at severity phase. Moreover, that TAF1L protein overexpression could promote the xenograft growth of OSCC was confirmed in nude mouse model. Taken together, this new finding suggested that the regulation of TAF1L protein expression can be as novel strategy for assisting early diagnosis and regulating OSCC process.

In this study, for investigating potential roles of TAF1L in OSCC via cell autophagy and apoptosis *in vitro* were at the next step. The levels of TAF1L protein expression were upregulated in two OSCC cell lines (Ca9-22 and Tca-8113), compared with that in normal oral epithelial cells (HOEC) and dysplastic oral keratinocytes (DOK) by Western blot. Furthermore, cell proliferation, migration, autophagy and apoptosis of both OSCC cell lines were modulated by siRNA-*TAF1L* treatment. Cell apoptosis and autophagy were obviously abnormalities in OSCC cells post *TAF1L* gene silence.

To avoid cell apoptosis is a typical characteristic in the most of cancers. Moreover, cell autophagy and apoptosis were pivotal regulation in the development of cancer, which could result in the severity of canceration 41,42. Increasing numbers of research has demonstrated that chemotherapeutic and phytochemical agents induced cell apoptosis was through reducing autophagy in tumors 43-46. As a potential protumor gene, *TAF1L* may have an important role as a regulator in response to the stresses for the apoptosis of cancer, which functions are similar to *TAF1* gene (its homologous family member) 20. Especially, it was rescued *TAF1L* gene knockdown-induced increase of apoptosis in OSCC cells, when autophagy activity is elevated. Taken together, the results showed that TAF1L could downregulate the apoptotic role for enhancing OSCC development via autophagic activation. The test indicates that inhibition of TAF1L protein expression may serve as potential therapeutic targets.

## Figures and Tables

**Table 1:** Clinical characteristics of OSCC patients obtained in this study

Parameters	Case numbers (n)	%
**Age (years)**		
≤50	37	61.82
>50	73	39.18
**Gender**		
Female	42	38.18
Male	68	61.82
**Histological differentiation**		
Well	81	73.64
Moderate	15	13.64
Poor	21	19.09
**Clinical stage**		
I	20	33.33
II	24	40
III	5	8.33
IV	1	1.67
Unknown	10	16.67
**T classification**		
T1	45	40.91
T2	43	39.09
T3	9	8.18
T4	11	10
Unknown	2	1.82
**Anatomic site**		
Tongue	68	61.82
Lip	13	11.82
Gingiva	7	6.36
Cheek	5	4.55
**Maxillary sinus**	4	3.64
Lower jaw	4	3.64
Upper jaw	3	2.73
Oral cavity	3	2.73
Palate	1	0.91
Parotid gland	1	0.91
Mandible	1	0.91

**Table 2:** TAF1L protein expression in OSCC tissues or normal oral/paracancerous tissues by IHC

	Case numbers (n)	Over-expression (n)	Over-expressive ratio (%)	*p*-value (Fisher's exact test)
**Normal tissues**	30	9	28.1%	0.0033*
**OSCC tissues**	110	96	87.3%	

* It means a statistically significant difference (*p* < 0.05).

**Table 3:** The association between TAF1L protein overexpression and clinicopathological characteristics of OSCC patients

Clinicopathological characteristics	TAF1L expression		
	Low (n)	High (n)	*p*-value
**Age (years)**			
≤50	13	44	0.6117
>50	10	43	
**Gender**			
Female	2	40	0.7079
Male	6	62	
**Histological differentiation**			
Well	4	77	0.1654
Moderate, poor	3	19	
**Clinical stage**			
I	8	4	0.2413
II-IV	51	27	
**T classification**			
T1	3	42	1
T2-4	57	58	
